# Editorial: Steroids and Secosteroids in the Modulation of Inflammation and Immunity

**DOI:** 10.3389/fimmu.2021.825577

**Published:** 2021-12-20

**Authors:** Andrzej T. Slominski, Bidesh Mahata, Chander Raman, Oxana Bereshchenko

**Affiliations:** ^1^ Department of Dermatology, University of Alabama at Birmingham, Birmingham, AL, United States; ^2^ Pathology Laboratory Service, Veteran Administration Medical Center, Birmingham, AL, United States; ^3^ Department of Pathology, University of Cambridge, Cambridge, United Kingdom; ^4^ Department of Philosophy, Social Sciences and Education, University of Perugia, Perugia, Italy

**Keywords:** steroids, secosteroids, steroidogenesis, secosteroidogenesis, Inflammation, immunity, vitamin D, glucocorticoids

In this Research Topic of Frontiers in Immunology focused on the steroids and secosteroids in the modulation of inflammation and immunity 11 articles by experts in corresponding fields have been published (Bier et al.; Bruscoli et al.; He et al.; König et al.; Lucafò et al.; Merk et al.; Postlethwaite et al.; Quatrini et al.; Shimba et al.; Vanderhaeghen et al.; Xie et al.). These included research articles, reviews and mini-reviews papers on important aspects of steroid- and secosteroidogenesis and the role of the final or intermediate products of these pathways in regulation of inflammatory and immune activities. Mechanisms of action and of broad homeostatic activities in humans and experimental animal models have also been discussed in these expert written papers. Different aspects of biochemistry, molecular biology, cell biology, and the systems-level role of (seco)steroidogenesis in regulating physiology and pathology have been discussed.

The key role in steroidogenesis is played by an enzyme CYP11A1, a member of the cytochrome P450 family, which catalyzes the first and rate-limiting step in steroidogenesis, converting cholesterol to pregnenolone through sequential its hydroxylation at C22 and C20, with a final cleavage of the side chain (1–4). In addition to the adrenals, gonads and placenta (classical steroidogenic tissues), CYP11A1 is also expressed in the brain (6), gastrointestinal tract, immune systems (7–9), the skin (5) and other peripheral organs/tissues (10) including malignant tumors (9, 11). The roles of local steroidogenesis (i.e., extra-glandular steroidogenesis, including immune cell mediated steroidogenesis) are emerging and warrant a revisit to this important biosynthetic pathway and its functional involvement in tissue homeostasis (including immune homeostasis) and disease (7, 12, 13). Recent discoveries pointed out an important, and unexpected role for CYP11A1 in metabolism of 7-dehydrocholestrol (14), vitamin D3 (15), D2 (16), lumisterol (17, 18) and ergosterol (11) to several biologically active metabolites. Thus, CYP11A1 activity is opening several novel pathways generating ∆7 steroids, full chain and short chain lumisterol derivatives, and secosteroids (vitamin D hydroxyderivatives) (5, 19). While the biological significance of these pathways is currently being evaluated, it must be noted that CYP11A1-derived hydroxyderivatives of vitamin D (20, 21) and of lumisterol (18) are circulating in human serum and are detectable in the epidermis. Furthermore, recent data showing that 7-Dehydrocholesterol reductase (DHCR7, which catalyzes the reduction of the C7-C8 double bond of its B-ring that is necessary for the final formation of cholesterol) did not act on 7-dehydropregnenolone and lumisterol compounds (22), enhances the importance of pathways generating ∆7 steroids and lumisterol derivatives (20, 23, 24).

As relates to secosteroids, in this Research Topic, a paper by Postlethwaite et al. reports that CYP11A1-derived 20S-hydroxyvitamin D3 [20S(OH)D3] markedly suppresses clinical signs of arthritis and joint damage in a mouse model of rheumatoid arthritis (RA). 20S(OH)D3 also changes proportion of lymphocyte subsets in peripheral blood resulting in a significant reduction in the levels of inflammatory cytokines, and decrease in complement-fixing anti-CII antibodies. The authors propose further consideration for 20S(OH)D3 in treatment of RA and other autoimmune disorders (Postlethwaite et al.).

Concerning extra-adrenal glucocorticoids synthesis, Merk et al. review a role of epithelial barriers as alternative routes for their synthesis at the local and perhaps systemic levels. Their role in the inter-organ communication through an interconnected crosstalk that counteract pro-inflammatory activities and prevent autoimmune activities are discussed (Merk et al.). These considerations are in line with similar concepts previously proposed (7, 13, 25, 26) and are consistent with communication between peripheral and central endocrine regulators (27) including hypothalamo-pituitary-adrenal axis (**Figure 1**) (7, 28, 29). Therefore, regulation of local corticosteroidogenesis may serve as a viable therapeutic alternative to using synthetic corticosteroids in the therapy of inflammatory or autoimmune disorders (Merk et al.; 7; 13). Such regulation can be achieved by using specific wavelength of ultraviolet radiation (UVR) (30).

**Figure 1:**
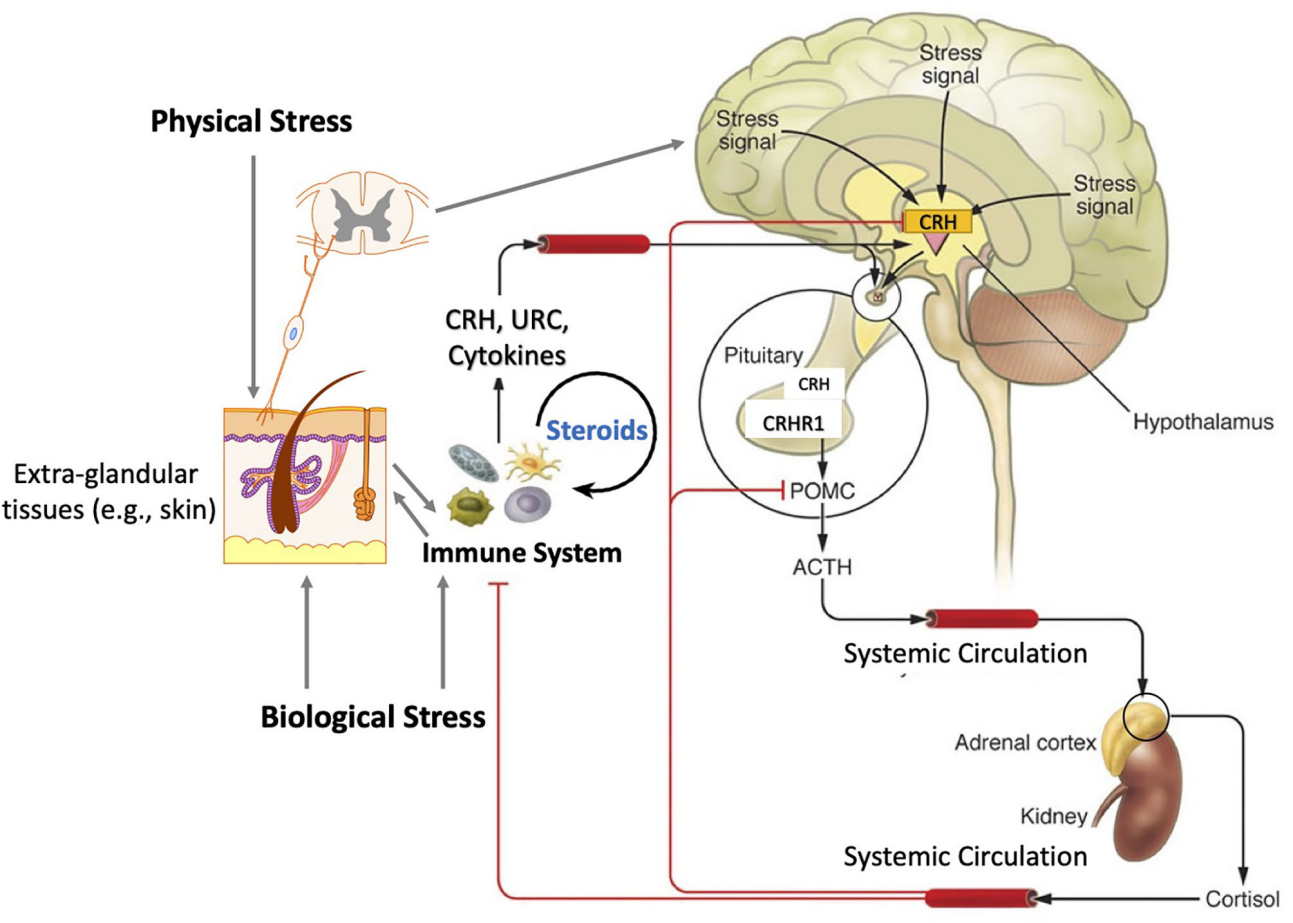
The functional organization of the hypothalamic-pituitary-adrenal axis with inputs from the immune system and peripheral organs. Physical and biological stress promotes the release of stress signals in both the brain, the skin, and immune cells, resulting in the hypothalamic release of corticotropin releasing hormone (CRH), which in turn stimulates the release of adrenocorticotropic hormone (ACTH) and pro-opiomelanocortin (POMC) expression and processing in the anterior pituitary. ACTH binds to the melanocortin-2 (MC2) receptor in the zona fasciculata of the adrenal cortex and stimulates the transport of cholesterol into the mitochondria and stimulates the production cortisol. Glucocorticoids not only regulate body homeostasis but also act in a negative feedback loop for CRH and POMC expression. Immune cell-mediated steroidogenesis and secosteroidogenesis are emerging as new modes of immune regulation. Reprinted with permission from the (7, 28).

Of similar importance are three other mini-reviews and two reviews. One on regulation of the immune system development by glucocorticoids and sex hormones (Quatrini et al.), which also include control of the hematopoietic stem cell differentiation and subsequent maturation of immune cell subsets. Second mini-review (Bruscoli et al.) discusses glucocorticoid therapy in inflammatory bowel diseases with consideration of new glucocorticoids mediators, such as glucocorticoid-induced leucine zipper, which may have similar anti-inflammatory properties. The third mini-review (Shimba et al.) discusses pleiotropic effects of glucocorticoids on the Immune system in the context of circadian rhythm and stress. Among two reviews one (He et al.) analyses glucocorticoid-induced leucine zipper as a promising marker for monitoring and treating sepsis. The second one (Vanderhaeghen et al.) discusses bidirectional crosstalk between hypoxia inducible factors and glucocorticoid signaling in health and disease, being in line with a fascinating subject of linking immune activity with immune cells energy yielding metabolism (31).

The four original research papers discuss important experimental evidences including use of IFNγ/IL10 ratio for stratification of hydrocortisone (a synthetic but identical molecule to endogenous cortisol) therapy in patients with septic shock (König et al.), glucocorticoid-induced exacerbation of mycobacterial infection through a reduced phagocytic capacity of macrophages (Xie et al.), protection of antigen-primed effector T cells from glucocorticoid-induced apoptosis in cell culture and in a mouse model of multiple sclerosis (Bier et al.), and that gender may influence the immunosuppressive actions of prednisone (a synthetic glucocorticoids with much higher potency than cortisol) in inflammatory bowel disease (Lucafò et al.).

Thus, this Research Topic discusses several important aspects of steroidogenesis, secosteroidogenesis, and their role in cell signaling cascades in the context of physiology and pathology of inflammation and immunity. One important conclusion is that the deregulation of steroidogenic and secosteroidogenic signaling pathways may also lead to a variety of inflammatory disorders and autoimmune diseases in a gender- and context-dependent manner. Different therapeutic and preventive strategies can be deducted from the presented papers leading to practical and clinical solutions to many inflammatory and autoimmune diseases. In future, further analytical investigation is required to understand the physiological and pathological role of endogenous steroids and secosteroids.

## Author Contributions

AS wrote the first-draft and others (BM, CR, OB) contributed to improve the writing and conceptual Figures. Correspondence can be made to anyone or all of these authors (AS, BM, CR, OB). All authors contributed to the article and approved the submitted version.

## Funding

Writing of this editorial was supported by NIH grants R01AR073004, R01AR071189, R21 AI149267, AR069010, AR064825, R21AR063242, VA merit grant 1I01BX004293-01A1. BM is supported by CRUK Career Development Fellowship (RCCFEL\100095), NSF-BIO/UKRI-BBSRC project grant (BB/V006126/1) and MRC project grant (MR/V028995/1).

## Conflict of Interest

The authors declare that the research was conducted in the absence of any commercial or financial relationships that could be construed as a potential conflict of interest.

## Publisher’s Note

All claims expressed in this article are solely those of the authors and do not necessarily represent those of their affiliated organizations, or those of the publisher, the editors and the reviewers. Any product that may be evaluated in this article, or claim that may be made by its manufacturer, is not guaranteed or endorsed by the publisher.
